# Cardiovascular Risk and Its Associated Factors in Health Care Workers in Colombia: A Study Protocol

**DOI:** 10.2196/resprot.4111

**Published:** 2015-07-30

**Authors:** Edna M Gamboa Delgado, Lyda Z Rojas Sánchez, Anderson Bermon Angarita, Yully Andrea Rangel Díaz, Silvia J Jaraba Suárez, Norma C Serrano Díaz, Evaristo Vega Fernández

**Affiliations:** ^1^ Fundación Cardiovascular de Colombia Research Center Fundación Cardiovascular de Colombia Floridablanca Colombia

**Keywords:** risk factors, metabolic syndrome, cardiovascular disease, prevalence, lifestyles

## Abstract

**Background:**

Cardiovascular diseases are the leading cause of mortality worldwide, for this reason, they are a public health problem. In Colombia, cardiovascular diseases are the main cause of mortality, having a death rate of 152 deaths per 100,000 population. There are 80% of these cardiovascular events that are considered avoidable.

**Objective:**

The objective of the study is to determine the prevalence of the cardiovascular risk and its associated factors among the institution’s workers in order to design and implement interventions in the work environment which may achieve a decrease in such risk.

**Methods:**

An analytical cross-sectional study was designed to determine the cardiovascular risk and its associated factors among workers of a high complexity health care institution. A self-applied survey will be conducted considering sociodemographic aspects, physical activity, diet, alcohol consumption, smoking, level of perceived stress, and personal and family history. In a second appointment, a physical examination will be made, as well as anthropometric measurements and blood pressure determination. Also, blood samples for evaluating total and high density lipoprotein cholesterol, triglycerides, and fasting blood sugar will be taken. A ten-year global risk for cardiovascular disease will be determined using the Framingham score. A descriptive analysis of the population’s characteristics and a stratified analysis by sex, age, and occupation will be made. Bivariate and multivariate analysis will be made using logistic regression models to evaluate the association between cardiovascular risk and the independent variables. The research protocol was approved by the Scientific and Technical Committee and the Ethics Committee on Research of the Fundación Cardiovascular de Colombia.

**Results:**

The protocol has already received funding and the enrollment phase will begin in the coming months.

**Conclusions:**

The results of this study will give the foundation for the design, implementation, and evaluation of a program based on promoting healthy lifestyles, such as performing regular physical activity and healthy food intake in order to avoid and/or control the cardiovascular risk in the workers of a high complexity health care institution.

## Introduction

### Cardiovascular Risk Factors

Cardiovascular risk factors are biological or behavioral characteristics that increase the probabilities of developing a cardiovascular disease (CVD) or dying from this cause [1], in those who have them. According to the World Health Organization (WHO), risk factors are classified in behavioral (modifiable) and biological. Behavioral risk factors include smoking, alcohol consumption, unhealthy diet, and physical inactivity, while the biological risk factors include hypertension, overweight, obesity, diabetes mellitus, and hypercholesterolemia [2].

The high prevalence of these risk factors has led CVD to become the leading cause of mortality worldwide, and, therefore, a public health problem. According to the WHO in 2008, these diseases were responsible for 30% of the mortality worldwide, almost 17.3 million people [3], from which 7.3 million were caused by coronary disease and 6.2 million by cerebrovascular disease [4]. In accordance with the WHO projections, the number of deaths by CVD worldwide will increase from 17 million in 2004 to 23.4 million in 2030 [5].

CVD affect not only the global mortality rates, but also the life quality of the population. CVD are responsible for 151.377 million disability-adjusted life years, from which 41.34% are due to coronary disease and 31% due to cerebrovascular disease [5]. Additionally, 90% of all deaths by CVD occur in low and medium income countries [6]. In contrast, it is estimated that 80% of these cardiovascular events are avoidable [7].

In the Latin-American population, the age adjusted mortality rate due to potentially treatable conditions is 42.2 for diabetes mellitus, 60 for heart ischemic disease, and 45.4 for cerebrovascular diseases per 100,000 population [8]. Studies conducted in this population have found a higher prevalence of risk factors in women than in men, except for smoking, which is higher between men [9].

In Colombia, CVD are the leading cause of death, being responsible for 28% of all deaths, and having a mortality rate of 152 deaths per 100,000 population. The main risk factors are overweight (48%) and physical inactivity (43%) [6,10]. In a regional context, in Santander-Colombia, the most prevalent cardiovascular risk factors are: low fruit/vegetable intake (94%) (less than 5 portions/day), low level of physical activity (70.6%), and overweight or obesity (50.7%) [11].

### Study of Health Care Workers in Bucaramanga, Santander

Otherwise, in a study conducted in health workers of a tertiary care institution in Bucaramanga, Santander, where the global cardiovascular risk and the prevalence of metabolic syndrome between workers were evaluated, a high prevalence of cardiovascular risk factors was determined, although the population was relatively young (median age was 44.3 years). In this study, the prevalence of hypertension was 54%, central obesity 40.3%, overweight 46.3%, obesity 21%, sedentary 82.4%, dyslipidemia 24%, smoking 10.4%, glucose intolerance 4.6%, and diabetes 1.6%. The ten-year global cardiovascular risk was 2.2% (5.2% in men and 1.4% in women). This study also established that male doctors were the population with the highest risk factors and the worst metabolic indices [12].

Fundación Cardiovascular de Colombia (FCV) is a high complexity health institution located in Floridablanca-Santander, Colombia. This institution is the biggest of the Colombian Northeast, and is specialized in the attention of CVD and their sequels. FCV’s health team is highly qualified and is dedicated to the prevention, diagnosis, and treatment of high complexity diseases, especially the cardiovascular ones.

Given the importance of maintaining a healthy lifestyle, FCV proposes the development of this study, with the objective of determining the prevalence of the cardiovascular risk and its associated factors among the institution’s workers in order to design and implement interventions in the work environment which may achieve a decrease in this risk.

## Methods

### Study Design

The design of the study is an analytical cross-sectional one.

### Study Participants and Eligibility

Workers from clinical and administrative areas of a high complexity health care institution specialized in the attention of CVD, of Floridablanca, Colombia (Fundación Cardiovascular de Colombia) will be included. No exclusion criteria will be considered.

### Sample Size

The institution has 1235 workers; 745 belong to the clinical area and 490 to the administrative area. All workers will be invited to participate in the study.

In order to increase the participation in the study, a sensitization strategy will be implemented by sending, through email, invitations and messages regarding the benefits of maintaining healthy lifestyles.

### Data Collection

**Figure 1:**
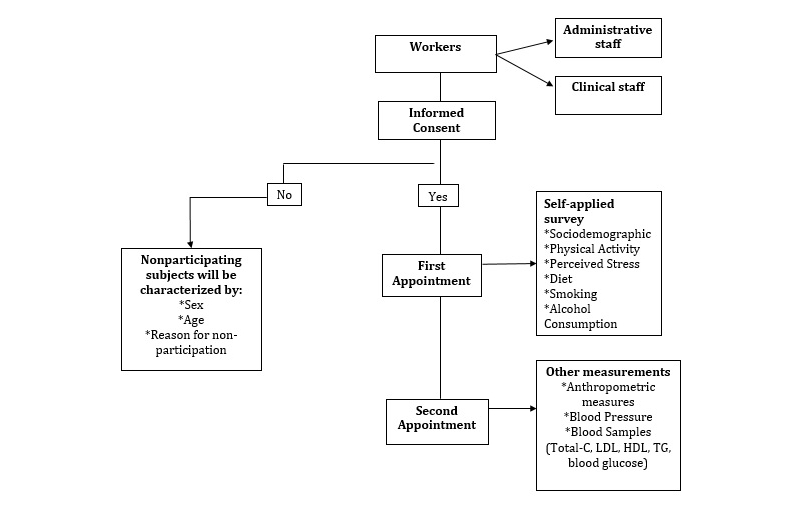
Data collection. Total-C: total cholesterol; LDL: low density lipoprotein; HDL: high density lipoprotein; and TG: triglycerides.

During 6 months, data collection will be held through two appointments. During the first appointment, a self-applied survey for sociodemographic aspects, physical activity, diet, alcohol consumption, smoking, level of perceived stress, and personal and family history of cardiovascular risk will be conducted with prior written informed consent. A second appointment will be made with the indication of wearing comfortable clothes and an 8-10 hours fasting, without having made intense physical activity or having consumed alcohol. Physical examinations will be made, and anthropometric measures and blood pressure will be recorded. Also, trained and qualified staff will take two blood samples; the first sample will assess total cholesterol levels, high density lipoprotein (HDL) cholesterol, triglycerides (TG), and blood glucose (Figure 1 shows this).

All personnel (nurses, physician, and nutritionist) involved in the research were previously trained on the procedures, tools, and instructional design for the collection of information, as well as for the quality control of data.

A second sample will be taken and stored in the FCV biobank in order to perform future measures of new biomarkers related to cardiovascular risk, such as homocysteine, tumor necrosis factor, and fibrinogen, which could be better predictors of cardiovascular risk than conventional markers [13].

### Study Variables

#### Sociodemographic Variables

Sociodemographic variables include age, sex, socioeconomic level, monthly income, level of education, marital status, social security, occupation, first-degree family history and personal history of cerebrovascular or CVD before age 60, reproductive history, cancer history, and whether or not the participant is receiving treatment for a chronic disease.

#### Smoking

A participant’s smoking habit will be measured using the smoking index, which includes the daily smoked cigarettes, the number of years the person has smoked, and an estimation of the total amount of cigarettes. It is calculated by the formula, (average number of cigarettes smoked per day) x (number of years smoked)/20 [14].

#### Alcohol Consumption

Alcohol consumption will be measured by the alcohol intake frequency questionnaire, which has been validated in Colombia [15]. Through this questionnaire, the frequency of alcohol consumption by specific alcoholic drink (beer, brandy, rum, wine, whisky) during the last month will be investigated.

#### Physical Activity Measurement

Physical activity will be evaluated using the International Physical Activity Questionnaire, short version, which was adapted to the Colombian population, considering the urban social context of low and middle socioeconomic strata, since they represent the highest proportion of people in the country [16-18]. The questionnaire evaluates the intense physical activity, the moderated physical activity, the weekly walking time, and the sitting time. The following categories will be considered for the analysis,

Category 1, or low, is for those who do not meet the criteria of the categories 2 and 3. They are considered inactive.

Category 2, or moderate, is for any of the following: 3 or more days of vigorous physical activity at least for 20 minutes a day; or 5 or more days of moderate-intense activity or walking for at least 30 minutes a day; or 5 or more days of any combination of walking, moderate-intense activity, or vigorous-intense activity achieving at least 600 minutes per week (MET).

Category 3, or high, is for either of the following two criteria: vigorous-intense activity for at least 3 days and accumulation of 1500 MET; or 7 or more days of any combination of walking, moderate-intense activity, or vigorous-intense activity achieving at least 3000 MET.

#### Diet Measurement

A questionnaire of frequency of food consumption during the last month will be given. This questionnaire will inquire about the most common food groups of the Colombian population. Also, the survey will inquire about some dietary habits.

#### Level of Perceived Stress

This variable will be measured through the perceived stress scale. Stress is a physical and psychological adaptive response to the demands and threats of the environment. The Perceived Stress Scale was designed with the purpose of discovering how stressful people perceive everyday life events. This scale is comprised of two dimensions: coping with stressors, and perception of stress [19,20].

This scale is composed of 14 items that assess how stressful the daily events in the last month are for people. This is a validated scale in the Colombian population, it has 5 answer options as in the Likert scale, where 0=never, 1=almost never, 2=occasionally 3=often, and 4= very often. The total score of the scale is obtained by inverting the scores of the items 4, 5, 6, 7, 9, 10, and 13 (as it follows, 0=4, 1=3, 2=2, 3=1, 4=0), and adding the 14 items. At a higher score, the higher is the level of perceived stress [19-21].

### Anthropometric Variables

#### Body Composition

The recommendations of the Manual of the International Society for the Advancement of Kinanth, the international standards for the anthropometric evaluation [22], will be applied.

#### Body Composition by Bioimpedance

The body composition analyzer Tanita TBF-300A will determine this. The scale will measure the following variables: weight, body fat percentage, fat body mass (kilogram, kg), lean body mass (kg), and total body water (kg).

#### Body Composition by Body Mass Index

This will be determined as the relation between the height and the body weight (kg/m2). The WHO defines a normal range of body mass index (BMI) as between 18.5 and 24.9; overweight is defined as a BMI >25, obesity is classified in 3 classes: class I with a BMI between 30-34.9; class II with a BMI between 35-39.9; and class III or morbid obesity with a BMI >40 [23,24].

#### Height

This will be measured in a 0.1 centimeter (cm) scale using a stadiometer. Subjects will be asked to remove their shoes and any head ornaments that may affect the measurement. The measurement will be read carefully, verifying that the squad of the stadiometer is stuck to the wall and horizontal to the plane of measurement. The reader’s eyes will be on the same horizontal plane as the stadiometer, in order to register an accurate measurement.

#### Waist Circumference

The measuring tape will be positioned at the smallest circumference of the natural waist or in the midpoint between the inferior margin of the last rib and the iliac crest, with the reader standing in front of the subject in order to localize correctly the measurement zone. Subjects will be asked to breathe normally and the measurement will be taken during expiration with the arms alongside the body. The cutoff point will be the one established by the International Diabetes Federation (IDF) for Latin population, ≥90 cm for men and ≥80 cm for women [25].

#### Hip Circumference

Subjects will be asked to stand up with feet together and without contracting the buttocks. Positioning the measuring tape around the maximum circumference of the buttocks and checking that the tape’s position is horizontal all around the body is how the perimeter will be measured.

#### Waist-Hip Ratio

This will be calculated by dividing the waist circumference (cm) by the hip circumference (cm). All the anthropometric variables will be measured twice, and the average of them will be taken for the analysis. The cutoff point will be, >0.90 for men and >0.85 for women and/or BMI >30 kg/m [26].

#### Blood Pressure

There are 3 readings that will be taken at intervals of at least two minutes, with a digital sphygmomanometer (Omron HEM-7114), having the following recommendations.

1. Subject's bladder must be empty, and they must have not smoked or drunk coffee 30 minutes before the measurement of the blood pressure (BP).
2. The patient should be seated comfortably for at least 5 minutes, with the back supported. The upper arm must be bared without constrictive clothing and supported at heart level. The legs should not be crossed and the feet must be on the floor.
3. The size of the cuff will be selected according to the circumference of the subject's arm (sizes S, M, L)
4. The brachial artery will be identified and the cuff bladder will be positioned 2 cm above the antecubital fossa.

BP ≥140 mmHg/90 mmHg [27] will be considered hypertension, as well as being in medical treatment for hypertension.

### Biochemical Variables

#### Blood Samples

Peripheral blood samples through venous puncture will be taken with the Vacutainer system, using three tubes of 7 ml without anticoagulant and two tubes of 4 ml with anticoagulant (Becton, Dickinson, and Co USA; Ref #366431yRef #366437, respectively). The samples will be centrifuged during 10 minutes at 3000 revolutions per minute; the component's separation will be made in the laboratory within a biological safety cabinet. The vials will be labeled with the assigned code and stored at -80°C. Prior to storage, the date, number of vials, and person responsible for the procedure will be recorded.

#### Fasting Blood Glucose

Fasting prior to sample taking will be verified, as well as pharmacological treatment with hypoglycemic drugs or insulin. Prediabetes will be considered with fasting blood glucose values between 100 milligrams per deciliter (mg/dl) and 125 mg/dl, and diabetes will be considered with fasting blood glucose ≥126 mg/dl (7.0 millimole per Liter, mmol/L), according to the American Diabetes Association [28].

#### Lipid Profile

Analysis of the lipid profile will be made by colorimetric techniques. The kits are in agreement with the ones referenced by the largest observational studies in CVD and include the following characteristics.

#### Total Cholesterol

The enzymatic method for quantitative determination of cholesterol was used. For analytic sensitivity, the method is linear up to 19.3 mmol/L (750 mg/dl); inferior limit of detection, 0.08 mmol/L (3 mg/dl). Hypercholesterolemia will be considered with cholesterol level >200 mg/dl [29].

#### Triacylglycerol

The colorimetric method was used to determine triacylglycerol levels. The analysis is linear up to 11.4 mmol/L (1000 mg/dl); inferior limit of detection, 0.05 mmol/L (4 mg/dl). Hypertriglyceridemia will be considered with values above 150 mg/dl [29].

#### High Density Lipoprotein Cholesterol

This was quantified by colorimetric assay (HDL-C plus) using esterase and cholesterol oxidase coupled to polyethylene glycol. The analytical sensitivity-inferior limit of detection: 0.08 mmol/L (3 mg/dl). Values will be considered abnormal if they are lower than 40 mg/dl for men and lower than 50 mg/dl for women [29].

#### Low Density Lipoprotein Cholesterol

This was calculated by the formula in Friedewald [30]. Low density lipoprotein (LDL) cholesterol (mg/dl)=total cholesterol-HDL cholesterol-triacylglycerol/5 or LDL cholesterol (mmol/L)=total cholesterol-HDL cholesterol-triacylglycerol/2.2. This formula will be applied when TG are ≤4.5 mmol/L. Abnormal values will be considered with LDL >130 mg/dl [29,31,32].

The methods were selected for being described, standardized, and analyzed according to the guidelines of the National Committee for Clinical Laboratory Standards manual EP5-T2 [33].

Metabolic syndrome will be defined by the Adult Treatment Panel III classification [29,33] and by the classification of the IDF [34,35].

#### Ten-Year Global Risk of Cardiovascular Disease

The risk (myocardial infarction, heart failure, angina, ischemic stroke, hemorrhagic stroke, transient ischemic attack, peripheral arterial disease) will be measured using the Framingham score, which contemplates the following variables: sex, age, systolic blood pressure, arterial hypertension in treatment, smoking, diabetes mellitus, HDL and total cholesterol, and determining four risk categories (low <15%, moderated 15%-20%, high 20%-30%, and very high >30%) [36].

#### Statistical Analysis

A descriptive analysis of the characteristics of the study population will be done. Continuous variables will be described with means and SD or median and interquartile range (25%-75%), according to their distribution assessed by the Shapiro-Wilk test. Categorical variables will be described as proportions with a 95% confidence interval.

Stratified analysis by sex, age, and occupation will be made. To identify the differences between comparison groups, Student *t* test will be used for continuous variables with normal distribution, and the Mann-Whitney test for continuous variables with skewed distribution. Categorical variables will be compared using the chi-square test or the Fisher´s exact test. For categorical variables, the comparison of proportions test will also be used. A stratified analysis by sex, age, and occupation will be made. Bivariate and multivariate analysis will be made using logistic regression models or lineal regression models, depending on the outcome variable, to evaluate the association between cardiovascular risk and the independent variables.

Additionally, an analysis of the basic data of the individuals who refuse to participate in the study (gender, age, reason for not participation) will be performed, and these data will be compared with those of the participants to minimize selection bias.

### Ethics and Dissemination

Research will be conducted in agreement with the 1993 Number 08430 Resolution from the Health Ministry of Colombia, by which the scientific, technical, and administrative standards for health research are established (Republic of Colombia, 1993) [37]. The Scientific and Technical Committee and the Ethics Committee on Research of the Fundación Cardiovascular de Colombia approved the research protocol. Written informed consent will be asked of all the participants of the study. The findings about this project will be disseminated through peer-reviewed publication and conference presentations.

## Results

Currently, this study is in the phase of training the personnel responsible for conducting the surveys and measurements. Data collection phase is expected to begin in two months. It is also expected that the data collection phase will end in a time lapse of sixth months given the total study population.

## Discussion

Given the interest of different institutions worldwide [38-41], which have emphasized the promotion of health in the workplace, this study will contribute evidence to occupational health by the establishment of the cardiovascular profile of the workers of a high complexity health care institute.

Determining the prevalence of the cardiovascular risk and its related factors will allow the design and assessment of the effectiveness of interventions directed to decrease or avoid those factors, which will comply with the WHO Strategy of Diet, Physical Activity, and Health. This strategy, established in article 64, “Workplaces are important environments for health promotion and disease prevention. People should be able to adopt health decisions in their workplace in order to reduce their exposure to risk. It is precise to ensure the opportunity of adopting healthy decisions in the workplace, as well as supporting and promoting physical activity” [42].

This study seeks to show the impact that generates an institutional program of promotion, prevention, and control of worker’s risk factors. Thus, this will support the objectives of the 60^th^ World Health Assembly, which highlights the global action plan on workers’ health 2008-2017 [43], where workplace and primary prevention of risk factors are considered the central focus for the economic development and world productivity.

## Abbreviations

BMI: body mass index
BP: blood pressure
cm: centimeter
COLCIENCIAS: Colombian Institute for the Development of Science and Technology
CVD: cardiovascular disease
FCV: Fundación Cardiovascular de Colombia
HDL: high density lipoprotein
IDF: International Diabetes Federation
kg: kilogram
LDL: low density lipoprotein
MET: minutes per week
mg/dl: milligrams per deciliter
mmol/L: millimole per liter
TG: triglycerides
WHO: World Health Organization
